# Can transthoracic echocardiography be used to a greater extent in the diagnostics of infective endocarditis to avoid unnecessary transoesophageal examinations without jeopardising accuracy?

**DOI:** 10.1186/s12947-023-00301-z

**Published:** 2023-01-31

**Authors:** Anna Damlin, Maria J. Eriksson, Eva Maret

**Affiliations:** 1 Department of Molecular Medicine and Surgery, Division of Clinical Physiology, Karolinska Institutet, (L1:00), Stockholm, 171 76 Sweden; 2 Department of Clinical Physiology, Karolinska University Hospital, Solna, A8:01, Eugeniavägen 3, Stockholm, SE-171 76 Sweden

**Keywords:** Infective endocarditis, Transoesophageal echocardiography, Transthoracic echocardiography

## Abstract

**Background:**

Infective endocarditis (IE) is a serious condition that requires prompt diagnosis and treatment. Transthoracic echocardiography (TTE) is usually the initial imaging modality, however transoesophageal echocardiography (TOE) is sometimes necessary because of its higher sensitivity for IE. Yet, TOE may imply an increased risk of complications. This project aims to evaluate whether TTE can be used to a greater extent in the diagnostics of IE to avoid unnecessary TOE examinations without jeopardizing diagnostic accuracy.

**Methods:**

Data from all TOE examinations performed on patients hospitalized with clinical suspicion of IE between 2019–05-01 and 2020–04-30 at a university hospital in Stockholm, Sweden, were obtained and analysed. Variables included for analysis were age, sex, blood culture results, aetiology, results from TOE, number of TOEs during the inclusion period, results from positron emission tomography/computed tomography (PET/CT), new regurgitation, cardiac murmur, previous IE, prosthetic valve, predisposing factors, i.e. cardiac comorbidities, injection drug use, fever, vascular phenomena, and immunological phenomena. To assess associations between predisposing factors or aetiology of IE and TOE findings, chi square tests and logistic regression models were used. For continuous variables, linear regression was used for comparisons of means and quantile regression was used for comparisons of medians. *P* < 0.05 was considered significant.

**Results:**

In total 195 TOE examinations (Table 1) from 160 patients were included, of which 61 (31%) were positive for IE. In total, 36 examinations had negative TTE prior to TOE of which 32 (86%) also had negative TOE. Of the 5 (14%) negative TTE prior to TOE that had positive TOE, all had cardiovascular implantable electronic device (CIED) and/or prosthetic valves.

**Conclusions:**

The existing recommendations for TOE in patients with clinical suspicion of IE are probably broad enough not to miss patients with IE, but there might be an unnecessarily large number of patients being referred for TOE with negative results. Negative TTE examination with good image quality and no CIED or prosthetic valves, may be sufficient without jeopardizing the IE diagnosis.

**Graphical Abstract:**

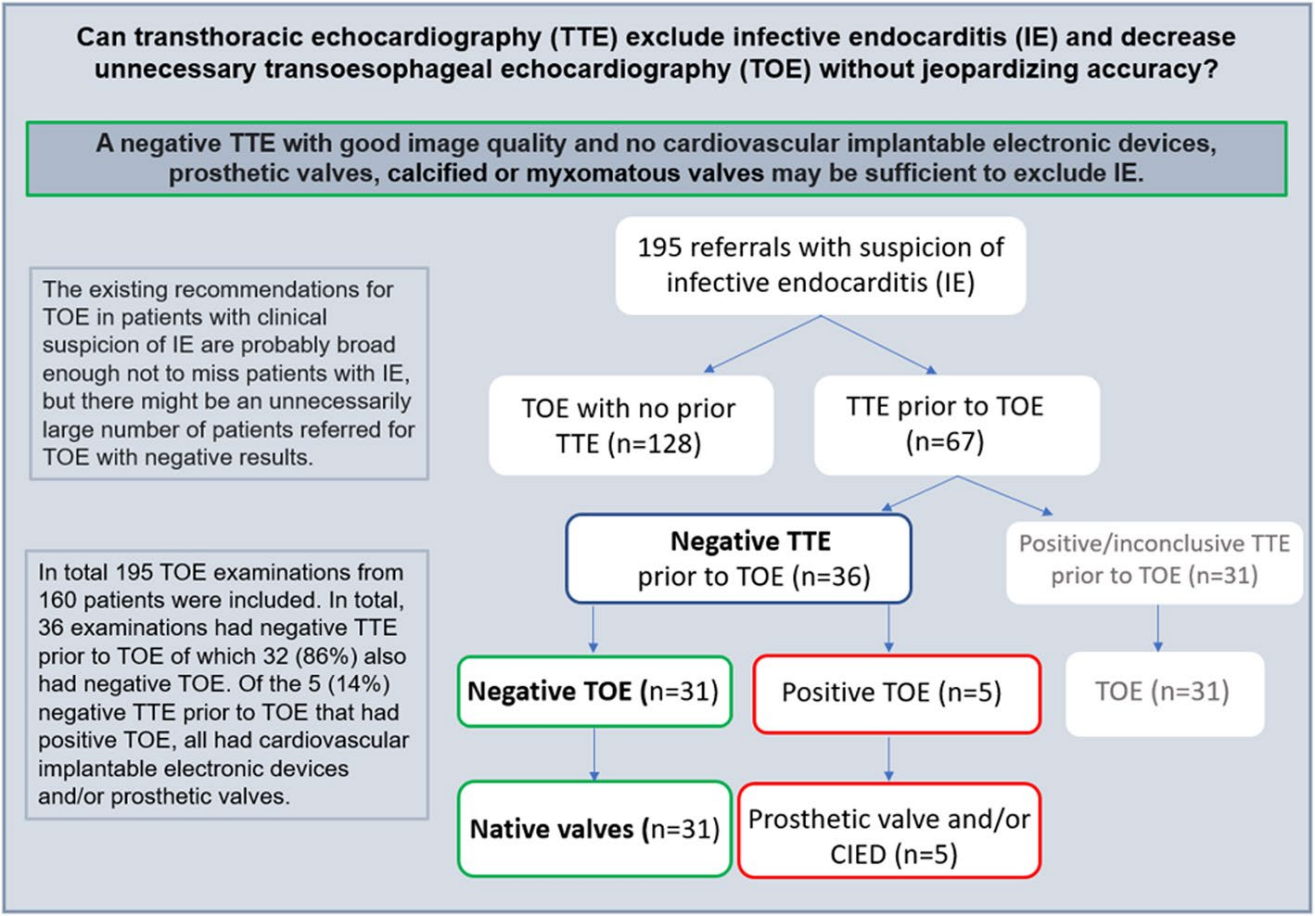

## Background

Infective endocarditis (IE) is one of the most common life-threatening infections, together with sepsis, pneumonia, and intra-abdominal abscess [1]. IE often causes vegetations on the heart valves, heart valve destructions, perivalvular abscesses, aneurysms or fistulas, therefore fast diagnosis and treatment are of great importance to avoid the progression of such manifestations [2, 3]. IE can rapidly be fatal if not accurately diagnosed and treated [4, 5]. In 2018, the mortality of patients with IE in Sweden was 13.6% [6]. Contrary to several other infectious diseases, the global burden of IE (measured in disability-adjusted life-years) is increasing [7].

IE is relatively rare in Sweden with an incidence of approximately 500 cases per year (i.e. 5 per 100 000 people) [6, 8, 9]. In current practice the diagnosis of IE is guided by the modified Duke criteria IE [2, 10]. According to the criteria, the diagnosis of IE can be defined as definite or possible [2, 10, 11]. According to the major criteria “imaging positive for IE”, echocardiography is the most used imaging technique [2]. Echocardiography can be performed as a transoesophageal (TOE) or transthoracic (TTE) examination [2]. Generally, TOE provides better image quality compared to TTE, and is therefore recommended for cases of prosthetic valves and intracardiac devices, or if a prior TTE had been negative but the clinical suspicion of IE is high [2, 12]. The sensitivity for the diagnosis of IE in native valves is 70% for TTE and 96% for TOE, while the sensitivity for the diagnosis of IE in prosthetic valves is 50% for TTE and 92% for TOE [13, 14]. The specificity for the diagnosis of IE has been reported as approximately 90% for both TTE and TOE. In summary, TOE has better sensitivity compared to TTE, both for native and prosthetic valves [15, 16].

Although the European and American guidelines for the management of endocarditis are clear, the adherence to the recommendations varies [2, 17, 18]. Sometimes, clinicians might refer the patient for a TTE when a TOE should be done according to guidelines, and sometimes patients are referred directly for a TOE although TTE would be sufficient for diagnosis or a better option, according to the risks associated with a TOE examination. The aim of this study was to evaluate the results from the echocardiographic examinations among patients with clinical suspicion of IE referred for TOE examination at a tertiary hospital (Karolinska University Hospital, Solna) in Stockholm, Sweden. The aim was further to assess the findings from the TOE examinations in relation to the indications for TOE according to the existing recommendations for echocardiographic management of IE, and to assess accuracy of a comprehensive TTE performed prior to TOE. The results could provide a basis for optimal use of TTE and TOE in the management of IE patients without jeopardizing the diagnosis for the patients with possible and defined IE.

## Methods

Data from all TOE referrals for examination to the Department of Clinical Physiology at Karolinska University Hospital in Solna, with the purpose to verify or exclude IE between 1 May 2019 and 30 April 2020 was obtained. Data was collected anonymized. Variables included for analysis were age, sex, blood culture results, aetiology, results from TOE, number of TOE examinations during the inclusion period, number of comprehensive TTE performed on separate days prior to TOE, results from positron emission tomography/computed tomography (PET/CT), new regurgitation, cardiac murmur, previous IE, prosthetic valve, cardiovascular implantable electronic device (CIED), predisposing factors, i.e., cardiac comorbidities, injection drug use, and fever, vascular phenomena, and immunological phenomena. If information was missing in the referrals for any of the variables, the variable was registered as missing value.

**Fig. 1:**
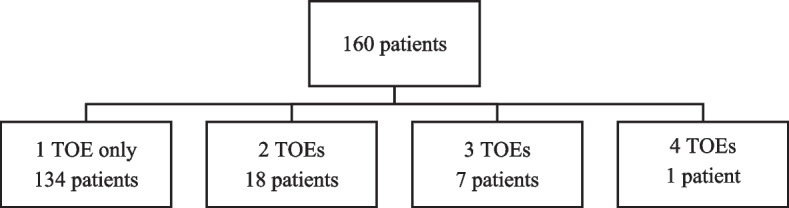
Number of transoesophageal echocardiography examinations performed for each patient with suspicion of infective endocarditis. Abbreviations: TOE, transoesophageal echocardiography

Most patients underwent one examination only, however, some patients underwent repeated TOE examinations due to continuous clinical suspicion of IE, or at suspicion of a new complication of IE (Fig. 1). Two patients had initial TOE examinations positive for IE, underwent surgical treatment for IE with prosthetic valve implantations and were then referred to follow-up TOE due to suspicion of a new complication of IE. One patient had an initial TOE negative for IE but due to continuous clinical suspicion of IE, a repeated TOE was conducted which was positive for IE (why the patient was surgically treated for IE) and then had a follow-up TOE.

### Statistical analysis

Continuous values were presented with means and standard deviations (SDs) or median and interquartile range (IQR), where applicable. Categorical values were presented with number and percentage. To assess associations between predisposing factors or aetiology and TOE findings, chi square tests and logistic regression models were used. For comparisons of continuous variables, linear regression was used for comparisons of means and quantile regression was used for comparisons of medians. *P*-values < 0.05 were considered statistically significant. The analyses were performed using STATA software (version 16 Stata Corp., College Station, Texas, USA).

## Results

### Characteristics of the study population

In total, 195 TOE examinations from 160 patients were included. The mean age was 60.0 years, with 42% females (Table 1). The female patients were younger (females 55.7 years vs. males 63.1 years, *P* < 0.01). The diagnosis of IE was established using the modified Duke criteria (Table 2). The most common bacteria from blood cultures were *Staphylococcus aureus* (*n* = 67, 34%), followed by streptococcus species (*n* = 18, 9%). In total, 22 (11%) of the patients presented with negative blood culture, and 34 (17%) with awaiting blood culture results prior to the TOE examination. Twenty-five patients (13%) underwent 18-fluorodeoxyglucose (FDG) PET/CT of which 4 (16%) were positive for IE, where the most common predisposing factor for IE among the PET/CT examinations was prosthetic heart valve (*n* = 44, 23%).

**Table 1:** Distribution of age, sex and the modified Duke criteria among patients with suspected infective endocarditis examined with transoesophageal echocardiography

**Patient characteristics**	**All patients**	**Patients with TOE positive for IE**
Number of patients, n (%)	160 (100)	44 (28)
Age, mean (SD), median (IQR)	60.0 (16.4); 63.0 (20.5)	58.7 (19.7); 64.5 (30.5)
Female, n (%)	68 (42)	19 (43)
Male, n (%)	92 (58)	25 (57)
Age of females, mean (SD); median (IQR)	55.7 (18.4)	51.2 (21.1); 55.0 (44.0)
Age of males, mean (SD); median (IQR)	63.1 (14.1)	65.9 (15.0); 72.0 (16.0)
**Clinical characteristics**	**All patients**	**Patients with TOE positive for IE**
Diabetes mellitus	5 (3)	3 (7)
Chronic kidney disease	8 (5)	5 (11)
Cancer	33 (21)	7 (16)
**Major Duke criteria**	**n (% of all patients)**	**n (% of patients with TOE positive for IE)**
Positive blood culture, n (%)	105 (66)	33 (75)
Gram positive bacteria	87 (54)	26 (59)
*Staphylococcus aureus*	48 (30)	16 (36)
Streptococci	8 (5)	2 (5)
CoNS	5 (3)	2 (5)
Candida	3 (2)	1 (2)
Enterococci	5 (3)	2 (5)
Negative blood culture, n (%)	22 (14)	4 (9)
Unknown result from blood culture, n (%)	33 (21)	7 (16)
Positive TOE, n (%)	44 (28)	
Aortic valve	15 (9)	
Mitral valve	23 (14)	
Tricuspid valve	2 (1)	
Pulmonary valve	1 (1)	
Dishiscence of prosthetic valve, n (%)	1 (1)	
Abscess, n (%)	2 (1)	
18FDG PET/CT, n (%)	21 (13)	8 (18)
18FDG PET/CT positive for IE, n (%)	4 (3)	1 (2)
**Minor criteria**		
Predisposing factors		
Intravenous drug use	5 (3)	2 (5)
Predisposing heart condition (excl. CIED and prosthetic valves)	4 (3)	4 (9)
CIED	4 (3)	3 (7)
Prosthetic valve (-s)	33 (21)	14 (32)
TAVR	14 (9)	6 (14)
bAVR	7 (4)	3 (7)
mAVR	5 (3)	1 (2)
bMVR	1 (1)	0 (0)
Pulmonary homograft	5 (3)	1 (2)
Freestyle aortic root	1 (1)	0 (0)
mAVR + mMVR	1 (1)	0 (0)
Fever (> 38 C)	97 (61)	31 (70)
Vascular phenomena, n (%)	20 (13)	7 (16)
Immunological phenomena, n (%)	0 (0)	0 (0)

*Abbreviations: bAVR* bioprosthetic aortic valve replacement, *bMVR* bioprosthetic mitral valve replacement, *CIED* Cardiovascular implantable electronic device, *CoNS* Coagulase negative staphylococci, *CT* Computed tomography, *FDG* Fluorodeoxyglucose, *IE* Infective endocarditis, *IQR* Interquartile range, *mAVR* mechanical aortic valve replacement, *mMVR* mechanical mitral valve replacement, *n* number, *PET* Positron emission tomography, *SD* Standard deviation, *TAVR* Transcatheter aortic valve replacement, *TOE* Transoesophageal echocardiography

**Table 2:** Presence of the modified Duke criteria among the TOE examinations

**Major criteria**	**All TOE examinations** **(n (%**^a^**))**	**Positive TOE examinations (n (%**^b^**))**
Positive blood culture, n (%)	137 (70)	49 (36)
Gram positive bacteria	115 (59)	42 (37)
*Staphylococcus aureus*	67 (34)	23 (34)
Streptococci	18 (9)	7 (38)
CoNS	13 (7)	4 (31)
Candida	5 (3)	2 (40)
Enterococci	5 (3)	2 (40)
Negative blood culture, n (%)	22 (11)	4 (18)
Unknown result from blood culture, n (%)	34 (17)	7 (21)
Positive TOE, n (%)	61 (31)	
Vegetation, n (%)	57 (29)	
Aortic valve	25 (13)	
Mitral valve	32 (16)	
Tricuspid valve	2 (1)	
Pulmonary valve	1 (1)	
Dishiscence of prosthetic valve, n (%)	1 (1)	
Abscess, n (%)	2 (1)	
18FDG PET/CT, n (%)	24 (12)	10 (42)
18FDG PET/CT positive for IE, n (%)	4 (2)	2 (50)
**Minor criteria**		
Predisposing factors		
Intravenous drug use	8 (4)	3 (38)
Predisposing heart condition (excl. CIED and prosthetic valves)	13 (7)	5 (38)
CIED	10 (5)	7 (70)
Prosthetic valve (-s)	44 (23)	17 (39)
TAVR	19 (10)	10 (59)
bAVR	9 (5)	3 (33)
mAVR	6 (3)	2 (33)
bMVR	1 (1)	1 (100)
Pulmonary homograft	6 (3)	1 (17)
Freestyle aortic root	1 (1)	0 (0)
mAVR + mMVR	1 (1)	0 (0)
Fever (> 38 C)	119 (61)	44 (37)
Vascular phenomena, n (%)	21 (11)	7 (33)
Immunological phenomena, n (%)	0 (0)	0 (0)

*Abbreviations: bAVR* bioprosthetic aortic valve replacement, *bMVR* bioprosthetic mitral valve replacement, *CIED* Cardiovascular implantable electronic device, *CoNS* Coagulase negative staphylococci, *CT* Computed tomography, *FDG* Fluorodeoxyglucose, *IE* Infective endocarditis, *mAVR* mechanical aortic valve replacement, *mMVR* mechanical mitral valve replacement, *PET* Positron emission tomography, *TAVR* Transcatheter aortic valve replacement, *TOE* Transoesophageal echocardiography

^a^ Percentage of the examinations for each row among all examinations

^b^ Percentage of the examinations with positive TOE among the examinations for each row. The information above is based on the examinations, not the patients, which means that if one patient underwent two (or more) TOE examinations positive for abscess, then two examinations positive for abscess was added to the table

### TTE results

About a third of all TOE examinations (*n* = 67, 34%), had a comprehensive TTE conducted on a separate day prior to the TOE examination. In total, 12 (18%) of the TTE examinations were positive for IE, 19 (28%) were inconclusive, and 36 (54%) were negative for IE. Of the 36 negative TTE examinations prior to TOE, 5 (14%) of the TOE examinations were positive for IE, of which all five had CIED and/or prosthetic valves. Among the examinations with a positive TTE prior to TOE, the TTE was conducted median (IQR) 5.0 (1.5–8.5) days prior to the TOE, 5.0 (3.0–9.0) days among those with an inconclusive TTE and 5.0 (3.0–10.0) days among those with a negative TTE prior to the TOE. Among the examinations with a negative TTE prior to a positive TOE, the TTE was conducted 6.0 (5.0–7.0) days prior to the TOE and among the examinations with a negative TTE prior to a negative TOE, the TTE was conducted 5.0 (2.0–7.0) days prior to the TOE. Possible reasons for the relatively long time between the TTE and TOE examinations that were not performed the same day were, initial low suspicion of IE, TOE was postponed due to patient-related or logistic reasons (contraindications for TOE i.e. high risk of bleeding, need for advanced sedation etc.), patients were transferred to our institution from another hospital where a TTE was performed, new suspicion of IE after removal of a CIED. All TTE and TOE examinations were assessed by at least two physicians specialized in echocardiography and cardiovascular imaging. In cases with uncertain results, a consensus was reached after discussion with another experienced specialist. Of the 19 inconclusive TTE examinations, 10 had too poor image quality to exclude or confirm IE, and 9 were considered inconclusive due to fibrotic elements, prosthetic valves, and calcifications.

### TOE indications and results

The indications for TOE according to the recommendations for echocardiographic management of IE are summarized in Table 3. In total, 159 (82%) examinations fulfilled any of the indications for TOE of which 57 (36%) had a positive TOE. The remaining 18% of the examinations were performed due to clinical suspicion of IE and awaiting blood culture results. The most common indication was “gram positive (incl. *S. aureus*) bacteraemia and clinical suspicion of IE” (*n* = 115, 59%) of which 42 (37%) had a positive TOE. In total 61 (31%) TOE examinations from 44 patients were positive for IE (Table 1), a significantly higher rate compared with the TTE examinations (12 (18%) positive examinations, *p* = 0.04). There was no significant difference in mean age among the patients with positive TOE compared to those with negative TOE (p (mean) = 0.92; p (median) = 0.32), as presented in Table 1. However, the female patients with positive TOE were significantly younger than the male patients with positive TOE (*p* < 0.01, Table 1). Mitral valve vegetation was the most common manifestation of IE identified by TOE (*n* = 32, 16%).

**Table 3:** Indications for TOE according to international recommendations for echocardiographic management of IE

**Indication for TOE**	**All TOE examinations** **(n (%))**	**Positive TOE examinations** **(n (%))**
Prosthetic valve IE, CIED, CVC associated infection	51 (26)	23 (45)
Suspicion of- or confirmed fungal IE	5 (3)	2 (40)
Gram positive (incl. *S. aureus*) bacteraemia and clinical suspicion of IE	115 (59)	42 (37)
Suspicion of abscess	2 (1)	2 (100)
Slow therapeutic response and persistent positive blood culture after 72–96 h	18 (9)	9 (50)
Complicated IE	10 (5)	10 (100)
Positive TTE	12 (6)	12 (100)
Inconclusive TTE and clinical suspicion of IE	19 (10)	6 (32)
Negative TTE and clinical suspicion of IE	36 (18)	5 (14)
Total	159 (82)	57 (36)

According to international recommendations [2, 13, 18], TOE should be performed in patients with at least one of the above listed indications

*Abbreviations: CIED* Cardiovascular implantable electronic device, *CVC* Central venous catheter, *IE* Infective endocarditis, *n* number, *TOE* Transoesophageal echocardiography, *TTE* Transthoracic echocardiography

## Discussion

Of the included 195 TOE examinations, about a third were positive for IE. Most of the examinations were conducted on basis of at least one clinical indication for TOE according to the current recommendations for diagnostic management of IE and of these, the majority had a negative TOE result. All the patients with native valves and no CIED, having a comprehensive TTE negative for IE performed prior to the TOE, also had a TOE negative for IE. These findings suggest that the existing recommendations for TOE in patients with clinical suspicion of IE are probably broad enough not to miss patients with IE, but there might be an unnecessarily large number of patients being referred for TOE with negative results. A negative TTE examination with good image quality and no CIED or prosthetic valves or other valve abnormalities, could possibly be sufficient without jeopardizing the IE diagnosis.

Among the examinations with inconclusive TTE prior to TOE and persistent clinical suspicion of IE, approximately a third of the TOE examinations were positive for IE. We therefore suggest that persistent clinical suspicion should lead to further diagnostic workup to establish the diagnosis. This strategy is recommended by the European Society of Cardiology guidelines for the management of IE [2]. Our study also emphasizes that good quality TTE is often sufficient to rule out IE in patients with native valves, however, an indeterminate TTE result should not be sufficient to exclude IE, as stated in the guidelines [2]. This is further supported by Bai et al., who suggests that a TTE with suboptimal view, or if any valvular abnormality is seen on TTE, a TOE should be performed even if no vegetation was visualized on the TTE [18].

The indication **“**prosthetic valve IE, CIED, or CVC associated infection” has been discussed in several guidelines [2, 13, 19]. As CIED and CVC devices can be located in anatomical areas only visible by TOE such as vena cava superior, TOE is considered indicated in these patients. Further, prosthetic valves and periprosthetic abnormalities are better visualized by TOE with better transmission and imaging options including recent development of three-dimensional (3D) imaging [2]. This is supported by a meta-analysis by Bai et al., suggesting TTE has low sensitivity for prosthetic valve vegetations and dehiscence, although TTE are often sufficient to exclude IE in patients with native valves as conclusively negative results on TTE greatly decrease the likelihood of IE [18]. Careful two-dimensional (2D) and Doppler TOE examination with addition of real-time 3D imaging enables visualization of the whole valve and surrounding structures, contributing to better assessment of vegetation morphology, and may overcome some imaging shortcomings of conventional TOE [19]. 3D echocardiographic imaging is also useful in the evaluation of perivalvular extension of IE, prosthetic valve dehiscence and perforation [20].

“TTE positive for IE” is one of the indications for TOE according to international recommendations for echocardiographic management of IE [2, 17, 21]. According to Rap et al., TOE is not required when TTE images are of good quality and there is no additional diagnostic yield [22]. However, TOE is useful in patients when the TTE image quality is poor or if the patients has a calcified aortic valve, myxomatous mitral valve or prosthetic valves [22]. Among the examinations with positive TTE prior to TOE, 100% of the TOEs were positive. Further, cases of complicated IE should routinely be examined with TOE to assess progression, abscess formations or prosthetic valve dehiscence, which are often better assessed with TOE. Multimodality approach with PET/CT should be considered for patients with suspicion of abscess, especially in cases with prosthetic valves [2].

Among the examinations with negative TTE prior to TOE and persistent clinical suspicion of IE, 5 examinations (14%) had a TOE examination positive for IE. All these five examinations had CIED, CVC or prosthetic heart valves-associated IE. This further strengthens **“**prosthetic valve IE, CIED or CVC associated infection” as an indication for TOE, also with a prior negative TTE.

The most common indication for TOE was “gram positive (incl. *S. aureus*) bacteraemia and clinical suspicion of IE”, comprising approximately 60% of the examinations. However, less than 40% of the examinations of patients fulfilling this indication had a positive TOE. All examinations of patients with gram positive (incl. *S. aureus*) bacteraemia and clinical suspicion of IE that underwent a TTE that was negative, had also a negative TOE examination. For patients with native valves, gram positive (incl. *S. aureus*) bacteraemia and clinical suspicion of IE, not having CIED or CVC, we suggest starting with a TTE examination, and if the image quality is good enough, these patients might not necessarily need a TOE. Among the examinations from patients with gram positive (incl. *S. aureus*) bacteraemia and clinical suspicion of IE, slow therapeutic response and persistent positive blood culture after 72–96 h, prosthetic valve, CIED, CVC, or injection drug use, were as commonly present in the examinations with positive TOE as in the examinations with negative TOE. Thus, slow therapeutic response, persistent positive blood culture after 72–96 h or injection drug use could indicate a renewal of a TTE examination but not mandatory a TOE. Some patients had negative blood cultures or pending blood culture results prior to the echocardiographic examination. We have noted that sometimes, the request of a TTE or TOE anticipates blood cultures results. This could be due to high clinical suspicion of IE (patient possibly fulfilling minor criteria) or if the patient is considered having higher risk of developing IE. This is in line with the guidelines, a patient with high clinical suspicion of IE should be early examined with echocardiography [2].

## Limitations

Data for this study were retrospectively collected from the referral system, not the medical record system, therefore crucial information could be missing in some referrals. There was not a substantially amount of missing data, of the 137 TOE examinations of patients with positive blood cultures, 7 (5%) did not have the bacteria species mentioned in the referral. Of the 195 referrals, 14 (7%) lacked information about new heart murmur, 13 (7%) lacked information about previous IE, and 35 (18%) lacked information about predisposing factors for IE. Notably, some of the referrals with missing information about possible predisposing factors for IE could have been patients with no predisposing factors for IE. This study focuses on the IE associated findings on TTE and TOE examinations, and we have not registered any additional information from the examinations, however it would have been interesting to assess any other discrepancies between TTE and TOE in order to validate TTE and TOE concordance, and to be able to assess if TOE added some additional clinically useful information. About a third of all the TOE examinations (*n* = 67, 34%), had a TTE conducted on a separate day prior to the TOE examination. Patients with initially high suspicion of IE and patients with prosthetic valves, calcified or myxomatous valves, CIED, or CVC are usually referred directly for TOE. These patients undergo a TTE just before the TOE (during the same examination session), however that TTE examination is primarily focused on the evaluation of left and right ventricular size and function, hemodynamic status, and not on detailed valve morphology. Some patients did not undergo TTE prior to TOE, these patients required general anesthesia to perform TOE or were treated in the intensive care unit with mechanical ventilatory support.

It should be noted that the TOE examinations in this study were conducted at a tertiary care, University hospital with access to top-of-the-line echocardiography equipment, advanced reconstruction software, and staff highly experienced in echocardiography. These factors constitute a possible limitation of the study, as other hospitals and health care centres might not have as advanced prerequisites of IE diagnostics. This may affect the generalizability of this study, as TTE examinations of poor quality is often not sufficient for the diagnosis of IE.

Further, this study was conducted using retrospective data from one hospital only (single centre study) which may affect its generalizability.

## Conclusions

A majority of the TOE examinations included in this study were negative for IE, however most of them had at least one indication for a TOE examination according to guidelines. The findings of our study suggest that the recommended indications for TOE are suitable to exclude IE but might implicate a risk of referring a relatively large number of patients not having IE for TOE examinations, as the majority of the examinations were negative for IE. TOE is useful in patients with intermediate to high suspicion of IE, when the TTE image quality is poor or if the patient has a calcified aortic valve, myxomatous mitral valve, prosthetic valve, CIED, or CVC, also in cases with gram positive (incl. *S. aureus*) bacteraemia. A negative TTE examination with good image quality and no CIED or prosthetic valves, could possibly be sufficient to exclude IE without jeopardizing the diagnosis.

## Data Availability

The data that support the findings of this study are available from the corresponding author on reasonable request.

## Abbreviations

CIED: Cardiovascular implantable electronic device
CVC: Central venous catheter
FDG: Fluorodeoxyglucose
IE: Infective endocarditis
IQR: Interquartile range
N: Number
PET/CT: Positron emission tomography/computed tomography
SD: Standard deviation
TOE: Transoesophageal echocardiography
TTE: Transthoracic echocardiography
